# Reply to Root-Bernstein: Increasing complexity allows for the pervasiveness of low-complexity entities and is not anthropocentric

**DOI:** 10.1073/pnas.2406598121

**Published:** 2024-08-12

**Authors:** Michael L. Wong, Stuart Bartlett, Carol E. Cleland, Heather Demarest, H. James Cleaves, Anirudh Prabhu, Jonathan I. Lunine, Robert M. Hazen

**Affiliations:** ^a^Earth and Planets Laboratory, Carnegie Institution for Science, Washington, DC 20015; ^b^NASA Hubble Fellowship Program Sagan Fellow, NASA Hubble Fellowship Program, Space Telescope Science Institute, Baltimore, MD 21218; ^c^Division of Geological and Planetary Sciences, California Institute of Technology, Pasadena, CA 91125; ^d^Department of Philosophy, University of Colorado Boulder, Boulder, CO 80309; ^e^Department of Chemistry, Howard University, Washington, DC 20059; ^f^Blue Marble Space Institute for Science, Seattle, WA 98104; ^g^Department of Astronomy, Cornell University, Ithaca, NY 14853; ^h^Cornell Center for Astrophysics and Planetary Science, Carl Sagan Institute, Cornell University, Ithaca, NY 14853

Root-Bernstein (1) argues that our usage of the terminology “selection for” (2) is not consistent with the picture of selection in evolutionary biology. We contend that this is a matter of semantics, for we agree that biological selection operates via diffuse, nonuniform survival/reproduction, and we do not invoke the requirement for “some agent” that drives nature toward a particular goal. Information is no more an agent of evolution than mass is an agent of gravity or entropy is an agent of the second law of thermodynamics; these are simply measurable parameters about the world that are useful for describing its regularities.

We contend that our current knowledge of living systems and complex systems (which include abiotic ones) is insufficient to support Root-Bernstein’s (1) claim that “the evolution of complexity is not different than the evolution of any given trait or species.” Complexity has many existing definitions and conceptual frameworks, unified by the idea that complexity is related to depth of description, i.e., the information content of the minimally sized and maximally predictive model or representation of a system (3). By any such metric, abiotic complexity on Earth has increased on average over time. A precise explanation for this observation remains elusive. Furthermore, we should not assume that the processes leading to long-term growth in complexity are exactly analogous to biological evolution by natural selection. In ref. 2, we argued for a generalized theory of selection and evolution that extends far beyond (but remains inclusive of) evolutionary biology.

Root-Bernstein writes, “The authors ignore low-complexity’s pervasive distribution, arguing that absent selection for complexity it may locally decrease, though information should still increase” (1). This is not an accurate representation of our proposal. Most of the universe could be said to be in a low-complexity state. Low-complexity systems persist for several reasons, among which is that some evolving systems, like atoms and minerals, appear to be bounded rather than open-ended. In bounded evolutionary systems, complexity may approach an asymptote with time.

Life is perhaps the only known evolving system to display true open-endedness (4, 5). We agree with Root-Bernstein that bacteria and archaea are extraordinarily successful. However, their apparent simplicity may be an artifact of viewing each cell in isolation. In reality, most single-celled organisms exist in collectives where nutrients are shared, functions are divided, intercellular communication is extensive, and resilience is fostered by cooperation (6, 7). Prokaryotes are arguably the most prevalent forms of life, but such prevalence is enabled by them being integrated within a diverse and complex system.

Finally, our model is not anthropocentric (1). Nowhere in our original paper did we state that humans are better examples of evolution than anything else or that there is any objective moral value to the presence of more functional information. All lifeforms on Earth have been selected by the environment as much as modern humans have. The functional information of Earth’s biosphere as a complex evolving system has clearly increased with time, from its emergence ~4 billion years ago to now.
